# Exploring Pathways Linking Work and Nonwork Factors to Sleep, Fatigue, and Health in Night Shift Nurses: A Structural Equation Modeling Analysis

**DOI:** 10.1155/jonm/6312917

**Published:** 2025-12-12

**Authors:** Bo Min Jeon, Su Hyun Kim

**Affiliations:** ^1^ College of Nursing, Susung University, Daegu, Republic of Korea; ^2^ College of Nursing, The Research Institute of Nursing Science, Kyungpook National University, Daegu, Republic of Korea, knu.ac.kr

**Keywords:** fatigue, health, nurse, shift work schedule, sleep–wake disorders

## Abstract

**Background:**

Night shift work disrupts sleep and physiological recovery, leading to adverse health outcomes among nurses. While both work and nonwork factors are known to influence sleep and fatigue, few studies have examined their simultaneous and multiple mediating effects on health in night shift nurses.

**Aim:**

This study tested a comprehensive model to investigate the relationships among work factors (emotional demands, night shift rotation intervals, and social support), nonwork factors (exercise and work–life interference), sleep disturbance, fatigue, and health in night shift nurses.

**Methods:**

A cross‐sectional study was conducted with 289 nurses working rotating night shifts at three hospitals in South Korea. Structural equation modeling (SEM) was employed to assess the direct and indirect effects between variables. Data were collected using validated instruments for sleep disturbance, fatigue, emotional demands, social support, exercise, work–life interference, and health.

**Results:**

Work–life interference had the most significant effect on health, both directly and indirectly through sleep disturbance and fatigue. Emotional demands negatively influenced health via sleep disturbance and fatigue. Exercise indirectly improved health by reducing fatigue, but showed no direct effects. Sleep disturbance was a significant predictor of both fatigue and health. Social support did not demonstrate significant direct or indirect effects on health. The final model explained 51.8% of the variance in health outcomes and exhibited strong model fit indices.

**Conclusion:**

Maintaining a work–life balance is critical for improving the health of night shift nurses, as work–life interference directly and indirectly exacerbates health risks through sleep disturbance and fatigue.

**Implications for Nursing Management:**

These findings highlight the importance of implementing strategies to manage work–life balance, address emotional demands, promote physical activity, and ensure recovery from shift work to support the health of night shift nurses.

## 1. Introduction

Night shift work is integral to healthcare, particularly for nurses, who represent the largest segment of the global healthcare workforce [1]. In many countries, including Korea, over 90% of hospital nurses operate under rotating three‐shift schedules [2]. In the United States, approximately 30% of nurses regularly work night shifts [3]. The disruption of circadian rhythms associated with night shifts leads to chronic sleep deprivation and fatigue [1], increasing the risk of various health conditions [4, 5]. This growing body of evidence underscores the urgent need to understand and mitigate the adverse health effects of night shift work, especially among nursing professionals [6].

Emerging research indicates that both work and nonwork factors directly and indirectly influence health outcomes [7]. Stressors inherent to night shift work—such as prolonged wakefulness, heavy workloads, and mental stress—worsen these outcomes, contributing to long‐term health deterioration [7–10]. Nonwork factors, including physical activity, social engagement, and work–life balance, may help counteract the adverse effects of night shifts by enhancing recovery and reducing psychological stress [11].

Previous studies have shown that work factors, such as job demands, schedule irregularities, and workplace hazards, are strongly linked to sleep disturbances and fatigue [8–10]. Social support and participation in restorative activities may stimulate the brain’s reward pathways by releasing neurotransmitters like dopamine and endorphins, thereby alleviating stress and fostering resilience [12]. However, empirical evidence regarding the interplay between these factors is limited, particularly in studies focused on nurses working night shifts. While numerous studies have documented the negative impacts of night shift work on health, significant gaps remain in understanding the complex interactions between work‐related stressors, nonwork factors, and health outcomes [7].

Given the complexity of these relationships, comprehensive models are necessary to integrate both work and nonwork factors, clarifying their cumulative and interactive effects on sleep, fatigue, and health. Developing such models is essential for informing evidence‐based interventions aimed at promoting the health and well‐being of nurses engaged in night shift work. The objective of this study is to test a theoretical framework that examines the direct and mediated pathways linking work and nonwork factors to sleep disturbances, fatigue, and broader health outcomes in nurses working night shifts. This research will provide critical insights into targeted strategies for improving occupational health and the quality of nursing care.

## 2. Materials and Methods

### 2.1. Study Design

This study employed structural equation modeling (SEM) to test a hypothesized model that examines the relationships among work and nonwork factors associated with night shift work, sleep disturbances, fatigue, and health outcomes. SEM was utilized to evaluate complex causal relationships and the mediating effects within a multivariate framework.

### 2.2. Conceptual Model

The study’s conceptual framework is based on Merkus et al.’s integrated theoretical model of nonstandard work schedules and health [7], which was further adapted based on empirical research [4, 13–17]. According to this model, work refers to the period when an individual invests mental and physical effort to perform job‐related tasks [7]. Nonwork pertains to the time dedicated to personal, familial, and social activities that promote recovery from night shift work.

**Figure 1:**
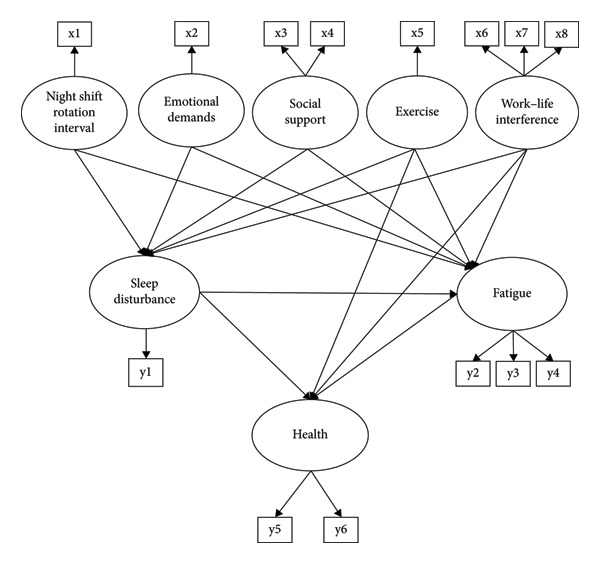
Hypothetical study model. *Note*: x1 = night shift rotation interval type; x2 = emotional demands; x3 = coworker support; x4 = supervisor support; x5 = average daily exercise; x6 = interference with leisure time; x7 = interference with domestic life; x8 = interference with nondomestic life; y1 = sleep disturbance; y2 = chronic fatigue item 1; y3 = chronic fatigue item 2; y4 = chronic fatigue item 3; y5 = physical health; y6 = mental health.

In the hypothesized model (Figure 1), work factors include night shift rotation intervals, emotional demands, and social support. Nonwork factors are represented by exercise and work–life interference. The study hypothesized that work factors (night shift rotation intervals, emotional demands, and social support) indirectly affect health, mediated by sleep disturbances and fatigue. Similarly, nonwork factors (exercise and work–life interference) were expected to influence health both directly and indirectly though the same mediating variables.

### 2.3. Study Population and Data Collection Procedures

This study conducted a secondary analysis using data from the project “Development of a Digital Healthcare Program to Promote Sleep in Shift Nurses.” The dataset included responses from 328 nurses working rotating night shifts at two university hospitals in City A and one general hospital in City B. In the participating hospitals, most nurses worked irregular rotating three‐shift schedules consisting of day, evening, and night shifts on an irregular rotating cycle without a fixed pattern. Data were collected between January 3, 2022, and May 12, 2022. The inclusion criteria were as follows: nurses (1) aged 19 or older, (2) working a three‐shift schedule that includes at least one night shift within the previous two weeks, and (3) able to read and communicate effectively in the study language. Nurses who were currently pregnant were excluded from participation. After applying the inclusion and exclusion criteria, 289 participants were retained for analysis. The adequacy of the sample size for SEM was assessed based on established guidelines. A minimum of 200 participants is generally recommended for SEM [18], with an alternative guideline suggesting 10 to 20 times the number of variables in the model [19]. Given that the structural equation model in this study included 14 variables, the sample size of 289 participants exceeded these thresholds, ensuring sufficient statistical power for model testing and estimation.

### 2.4. Measurements

#### 2.4.1. Endogenous Variables

Sleep disturbance was measured using the Sleep Disturbance Scale from the Survey of Shiftworkers [20]. This scale consists of 25 items addressing various aspects of sleep quality, including adequate sleep, good sleep, recovery after sleep, waking early, and difficulty falling asleep. Responses are recorded on a 5‐point Likert scale, ranging from 1 (“almost never”) to 5 (“almost always”), with a total score ranging from 25 to 125. Higher scores indicate more severe sleep disturbances. The scale demonstrated good reliability, with a Cronbach’s alpha of 0.87 in a previous study conducted among nurses [21] and 0.80 in this study.

Fatigue was measured using the 3‐item Chronic Fatigue Scale from the Survey of Shiftworkers [20]. Each item was rated on a 5‐point Likert scale ranging from 1 (“not at all”) to 5 (“very much so”). Total scores ranged from 3 to 15, with higher scores indicating greater severity of chronic fatigue. The scale demonstrated acceptable reliability, with a Cronbach’s alpha of 0.72 in a previous study of workers on two‐ and three‐shift schedules [22].

Health outcomes were measured using the physical and mental health subscales from the Survey of Shiftworkers [20]. The physical health subscale consists of 20 items addressing digestive symptoms, cardiovascular symptoms, and musculoskeletal pain, rated on a 4‐point Likert scale from 1 (“almost never”) to 4 (“almost always”). Total score ranges from 20 to 80, with higher scores indicating poorer physical health. Reliability was high, with Cronbach’s alpha values ranging from 0.85 to 0.86 in previous studies [22] and 0.87 in this study. The mental health subscale includes 12 items rated on a 4‐point Likert scale ranging from 0 (“not at all”) to 3 (“very much so”), with total scores ranging from 0 to 36. Higher scores indicate poorer mental health. Cronbach’s alpha was 0.86 in a previous study [22] and 0.87 in this study.

#### 2.4.2. Work Factors

Night shift rotation interval was defined as the number of days between the end of one night shift and the beginning of the next scheduled night shift, including any intervening off‐duty, days, or evening shifts. Night shift rotation intervals were assessed using a single‐item measure from the Survey of Shiftworkers [20]. Participants were asked to report the number of days between their last night shift and the next scheduled night shift over 2 weeks. Participants were categorized into short‐ or long‐rotation interval groups based on the median value.

Emotional demands during work were measured with two items adapted from the 5th Korean Working Condition Survey [23], developed based on the European Work Condition Survey [24]. Items were scored on a 7‐point scale from 1 (“all the time”) to 7 (“never”). This study converted scores to a 100‐point scale, with higher scores indicating greater emotional demands. Cronbach’s alpha was reported as 0.87 in the European Working Environment Survey [24] and 0.62 in this study.

Social support was measured using two items from the 5th Korean Working Condition Survey [23], assessing support from coworkers and supervisors. Each item was rated on a 5‐point Likert scale ranging from 1 (“always”) to 5 (“never”). This study reverse‐scaled and converted scores to a 100‐point scale, with higher scores indicating greater workplace social support. Cronbach’s alpha values for this instrument ranged from 0.70 to 0.79 in previous studies [24].

#### 2.4.3. Nonwork Factors

Exercise was assessed by asking participants to report their average daily minutes of moderate‐to‐intense physical activity outside of work over 14 days. Work–life interference was measured using the three‐item Social and Family Life Interference Scale from the Survey of Shiftworkers [20]. The scale includes items addressing interference with leisure time, domestic life, and nondomestic life, each rated on a 5‐point Likert scale ranging from 1 (“not at all”) to 5 (“very much so”). Total scores range from 3 to 15, with higher scores indicating more significant work–life interference. The scale’s reliability was strong, with a Cronbach’s alpha of 0.88 reported in a previous study of nurses [21].

### 2.5. Data Analysis

Descriptive statistics were used to summarize the participants’ general characteristics and study variables. The assumption of multivariate normality was assessed using Mardia’s multivariate skewness and kurtosis coefficients [25]. The analysis revealed that the assumption was violated, with a kurtosis coefficient of 26.01 (critical ratio = 10.44). Given the violation of normality, SEM was conducted using a maximum likelihood estimation with bootstrapping. Bootstrapping was employed to assess the statistical significance of indirect effects. User‐defined estimates were calculated to evaluate specific pathways within the model. Model fit was assessed using multiple indices, including the chi‐square divided by degrees of freedom (*χ* ^2^/df), goodness of fit index (GFI), adjusted goodness of fit index (AGFI), comparative fit index (CFI), root mean square error of approximation (RMSEA), and standardized root mean square residual (SRMR). All analyses were performed using SPSS Version 26.0 (IBM Corp., Armonk, NY, USA) and AMOS Version 26.0 (IBM Corp., Armonk, NY, USA).

### 2.6. Ethical Considerations

The Institutional Review Board (IRB) of Kyungpook National University approved this study (IRB File No. 2022‐0320). Voluntary written informed consent was obtained from all participants prior to participation. All procedures involving human participants were conducted in compliance with relevant guidelines and regulations, including the Declaration of Helsinki. To protect participant confidentiality, all personal data were anonymized and stored securely, with access limited to authorized research personnel only.

## 3. Results

### 3.1. Participants’ Characteristics and Study Variables

As shown in Table 1, most participants were female (*n* = 273, 94.5%), with a mean age of 27.35 ± 3.82 years. Most participants were single (*n* = 253, 87.5%) and worked in general wards (*n* = 188, 65.1%), followed by intensive care units (*n* = 66, 22.8%) and emergency departments (*n* = 30, 10.4%). The average length of nursing experience was 52.76 ± 43.00 months.

**Table 1:** Participant characteristics (*N*  = 289).

Variables	*n* (%)/M ± SD	Min–max
Gender		
Male	16 (5.5)	
Female	273 (94.5)	
Age (years)	27.35 ± 3.82	23–46
Marital status		
Single	253 (87.5)	
Married	36 (12.5)	
Living with children		
Yes	14 (4.8)	
No	275 (95.2)	
Number of children		
1	4 (28.6)	
2	10 (71.4)	
Average age of children (years)	5.36 ± 3.34	0–13.50
Work department		
Ward	188 (65.1)	
ICU	66 (22.8)	
Emergency room	30 (10.4)	
Operation room	5 (1.7)	
Clinical career (months)	52.76 ± 43.00	3–264
Shift pattern		
Irregular rotating three‐shift	289 (100.0)	
Consecutive night shifts	2.24 ± 0.46	2–4
2	223 (77.2)	
3	62 (21.4)	
4	4 (1.4)	
Number of night shifts per month	3.85 ± 1.06	2–8
2	33 (11.4)	
3	52 (18.0)	
≥ 4	204 (70.6)	
Night shift rotation interval (days)	7.25 ± 3.64	1–12
Short (< 9)	106 (36.7)	
Long (≥ 9)	183 (63.3)	
Emotional demands	47.61 ± 21.80	8.30–100
Social support	68.81 ± 15.77	12.50–100
Coworker support	75.69 ± 14.57	25–100
Supervisor support	61.94 ± 21.95	0–100
Exercise (min/day)	14.24 ± 19.70	0–110.77
Work–life interference	8.38 ± 2.51	3–15
Interference with leisure time	3.13 ± 0.93	1–5
Interference with domestic life	2.55 ± 1.05	1–5
Interference with nondomestic life	2.70 ± 1.10	1–5
Sleep disturbance	69.74 ± 12.12	39–100
Fatigue	9.42 ± 2.14	4–15
Physical health	37.55 ± 8.35	22–65
Mental health	12.22 ± 4.88	4–32

*Note:* M, mean.

Abbreviation: SD, standard deviation.

All participants worked irregular rotating three‐shift schedules consisting of day (07:00–15:00), evening (15:00–23:00), and night (23:00–07:00) shifts. Most participants worked two (*n* = 223, 77.2%) or three (*n* = 62, 21.4%) consecutive night shifts, while only a few (*n* = 4, 1.4%) reported working four consecutive nights. In addition, the majority (70.6%, *n* = 204) reported working more than four night shifts per month. The mean interval between night shift was 7.25 ± 3.64 days (median = 9 days), and approximately two‐thirds of participants were categorized into the long‐rotation interval group (≥ 9 days) for analysis.

The mean score for emotional demands was 47.61 ± 21.80. Social support was higher from coworkers (75.69 ± 14.57) than supervisors (61.94 ± 21.95). Participants engaged in an average of 14.24 ± 19.70 min of exercise per day. Work–life interferences were highest for leisure time (3.13 ± 0.93), followed by nondomestic life (2.70 ± 1.10) and domestic life (2.55 ± 1.05). The mean scores for sleep disturbance, fatigue, physical health, and mental health were 69.74 ± 12.12, 9.42 ± 2.14, 37.55 ± 8.35, and 12.22 ± 4.88, respectively.

### 3.2. Correlations Among the Study Variables

The results of the correlational analysis revealed that the mental health score was positively associated with sleep disturbance, fatigue, emotional demands, and work–life interference, including leisure time, domestic life, and nondomestic life (*r* = 0.15 to 0.32, *p* < 0.05 for all). Conversely, the mental health score was negatively correlated with coworker support, supervisor support, and exercise (*r* = −0.16 to −0.23, *p* <  .05). The physical health score was positively correlated with sleep disturbance, fatigue, emotional demands, and work–life interference (*r* = 0.13 to 0.28, *p* < 0.05) and negatively associated with exercise (*r* = −0.16, *p* < 0.05). All correlation coefficients were below 0.70, indicating no multicollinearity.

### 3.3. Measurement Model

Table 2 summarizes the results of the confirmatory factor analysis. Construct reliability (CR) values ranged from 0.74 to 0.85, standardized factor loadings ranged from 0.50 to 0.90, and average variance extracted (AVE) values ranged from 0.50 to 0.74. These values met the criteria for convergent validity, which include (a) CR ≥ 0.70, (b) standardized factor loadings (*λ*) ≥ 0.50, and (c) AVE ≥ 0.50 [26].

**Table 2:** Convergent validity of the measurement model.

Latent variable	Observed variable	Regression weights	S.E	C.R.	AVE	CR
Night shift rotation interval	x1	1	—	—	1	1

Emotional demands	x2	1	—	—	1	1

Social support	x3	0.64	—	—	0.63	0.77
	x4	0.73	0.41	4.23^∗∗^		

Exercise	x5	1	—	—	1	1

Work–life interference	x6	0.82	—	—	0.50	0.74
	x7	0.74	0.11	9.65^∗∗^		
	x8	0.57	0.10	8.26^∗∗^		

Sleep disturbance	y1	1	—	—	1	1

Fatigue	y2	0.90	—	—	0.59	0.81
	y3	0.61	0.08	8.83^∗∗^		
	y4	0.64	0.08	9.14^∗∗^		

Health	y5	0.50	—	—	0.74	0.85
	y6	0.67	0.26	5.42^∗∗^		

Abbreviations: AVE, average variance extracted; C.R., critical ratio; CR, construct reliability; S.E, standard error.

^∗∗^ *p* < 0.001.

### 3.4. Structural Model: Hypothesized Model Testing and Modification

**Figure 2:**
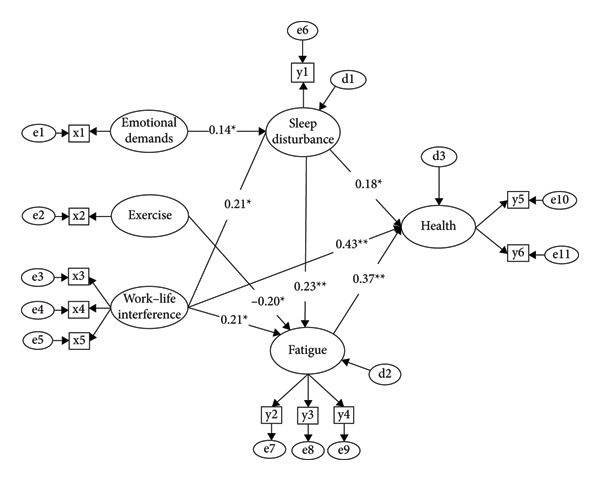
Path diagram of the modified model. *Note*: x1 = emotional demands; x2 = average daily exercise; x3 = interference with leisure time; x4 = interference with domestic life; x5 = interference with nondomestic life; y1 = sleep disturbance; y2 = chronic fatigue item 1; y3 = chronic fatigue item 2; y4 = chronic fatigue item 3; y5 = physical health; y6 = mental health. ^∗^ *p* < 0.05; ^∗∗^ *p* < 0.001.

The initial hypothesized model demonstrated an acceptable fit with the following indices: *χ* ^2^/df = 2.33, GFI = 0.94, AGFI = 0.89, CFI = 0.90, SRMR = 0.06, and RMSEA = 0.07. Of the 17 hypothesized paths, seven were statistically significant. To improve model parsimony and fit, statistically insignificant paths were removed, including the paths from night shift rotation intervals to fatigue and sleep disturbance, emotional demands to fatigue, social support to sleep disturbance and fatigue, and exercise to sleep disturbance and health. The refined model exhibited improved fit: *χ* ^2^/df = 2.44, GFI = 0.95, AGFI = 0.91, CFI = 0.92, SRMR = 0.06, and RMSEA = 0.07. A chi‐square difference test (Δ*χ* ^2^/df = 42.76/20, *p* < 0.001) confirmed that the revised model achieved better parsimony while maintaining good overall fit (Figure 2).

### 3.5. Direct, Indirect, and Total Effects of Key Variables on Health in the Final Model

Table 3 presents the results of the mediation analysis, including the direct, indirect, and total effects of key variables on health. Among the total effects, work–life interference had the largest influence on health (*β* = 0.56, *p* = 0.001), followed by fatigue (*β* = 0.37, *p* = 0.001) and sleep disturbance (*β* = 0.26, *p* = 0.008). Work–life interference showed significant direct and indirect effects on health (*β* = 0.43, *p* = 0.001; *β* = 0.13, *p* = 0.002, respectively). Both emotional demands and exercise also exhibited significant total effects on health through indirect pathways (*β* = 0.04, *p* = 0.011; *β* = −0.07, *p* = 0.001, respectively). The final model accounted for 51.8% of the variance in health outcomes.

**Table 3:** Standardized direct, indirect, and total effects of the modified model.

Endogenous variables	Exogenous variables	Direct effect (*p*)	Indirect effect (*p*)	Total effect (*p*)
Sleep disturbance	Emotional demands	0.14 (0.010)	—	0.14 (0.010)
	Work–life interference	0.21 (0.008)	—	0.21 (0.008)

Fatigue	Emotional demands	—	0.03 (0.006)	0.03 (0.006)
	Exercise	−0.20 (0.001)	—	−0.20 (0.001)
	Work–life interference	0.21 (0.009)	0.05 (0.006)	0.26 (0.001)
	Sleep disturbance	0.23 (0.002)	—	0.23 (0.002)

Health	Emotional demands	—	0.04 (0.011)	0.04 (0.011)
	Exercise	—	−0.07 (0.001)	−0.07 (0.001)
	Work–life interference	0.43 (0.001)	0.13 (0.002)	0.56 (0.001)
	Sleep disturbance	0.18 (0.030)	0.09 (0.001)	0.26 (0.008)
	Fatigue	0.37 (0.001)	—	0.37 (0.001)

Work–life interference was the strongest direct predictor of sleep disturbance (*β* = 0.21, *p* = 0.008), followed by emotional demands (*β* = 0.14, *p* = 0.010). The model explained 7.6% of the variance in sleep disturbance. Work–life interference also had the largest total effect on fatigue (*β* = 0.26, *p* = 0.001), followed by sleep disturbance (*β* = 0.23, *p* = 0.002), exercise (*β* = −0.20, *p* = 0.001), and emotional demands (*β* = 0.03, *p* = 0.006). The model explained 17.2% of the variance in fatigue.

The mediation analysis revealed several significant indirect effects. Sleep disturbance mediated the relationships between emotional demands and health (*b* = 0.004, 95% CI = 0.001–0.012) and between work–life interference and health (*b* = 0.010, 95% CI = 0.001–0.030). Fatigue significantly mediated the effects of exercise (*b* = −0.001, 95% CI = −0.002 to −0.001) and work–life interference (*b* = 0.021, 95% CI = 0.004–0.063) on health. Additionally, there were multiple mediation effects in the pathways from emotional demands through sleep disturbance to fatigue on health (*b* = 0.002, 95% CI = 0.001–0.006) and from work–life interference through sleep disturbance to fatigue on health (*b* = 0.005, 95% CI = 0.001–0.015).

## 4. Discussion

This study model elucidates the interplay of work and nonwork factors and their effects on sleep disturbance, fatigue, and health among night shift nurses. The findings provide empirical evidence to support interventions aimed at improving the health and well‐being of night shift nurses by addressing emotional demands, promoting exercise, and mitigating work–life interference. The model demonstrated a strong fit and substantial explanatory power, accounting for a significant proportion of variance in the key study variables. Additionally, all hypothesized paths were statistically significant, and the 95% confidence intervals excluded zero. The observed standardized effects were small to moderate in magnitude, supporting the theoretical framework of this study. To our knowledge, this is the first study to examine the simultaneous and multiple mediating effects of work and nonwork factors on sleep, fatigue, and health in night shift nurses.

First, the findings indicate that the nonwork factors had a greater impact on health outcomes than work factors. Specifically, work–life interference emerged as the strongest predictor of health, demonstrating both direct and indirect effects through the single and multiple mediating pathways of sleep disturbance and fatigue. Severe work–life interference directly impairs and deteriorates health by disrupting sleep quality and contributing to fatigue. These findings clarify the mechanisms through which leisure activities and personal life disruptions influence sleep quality, fatigue [27, 28], health‐related behaviors, and health among shift workers [29, 30]. The direct effect of work–life interference on health (*β* = 0.43, *p* = 0.001) was stronger than its indirect effects (*β* = 0.13, *p* = 0.002), highlighting the independent impact of maintaining a work–life balance. This suggests that work–life interference alone can significantly impair health, even in the absence of sleep disturbances and fatigue. Therefore, targeted interventions to support work–life balance are essential for reducing the health risks associated with night shift work [31].

Furthermore, exercise indirectly affected health by reducing fatigue, but did not have a direct effect. This suggests that regular exercise alleviates work‐related fatigue by enhancing physiological recovery, indirectly improving health outcomes. These findings align with previous research showing that leisure‐time physical activity reduces fatigue among shift workers [32] and that lifestyle behaviors, such as exercise and weight management, influence health indirectly through fatigue reduction in night shift nurses [33]. The beneficial effects of exercise may be mediated by neurophysiological mechanisms, including the release of neurotransmitters that enhance stress resilience and recovery [14]. However, the absence of a direct association between exercise and health may be due to the relatively young age of participants (mean = 27 years) and their shorter duration of exposure to shift work, which may not yet have led to the development of chronic health conditions [34].

Interestingly, social support from coworkers and supervisors did not have significant direct or indirect effects on health. This finding contrasts with previous research suggesting that social support protects against sleep disturbances and fatigue [8, 9], but aligns with studies reporting no significant associations [35]. This inconsistency may be attributable to the measurement of social support in the present study, which included only coworker and supervisor support but not family support—a crucial source of resilience for night shift nurses [36]. The omission of family support highlights the need for caution in interpreting the results. Future studies should incorporate both workplace and family support to more comprehensively examine the buffering effects of social resources on health outcomes.

Second, regarding work factors, emotional demands indirectly influence health via sleep disturbance and fatigue, disrupting sleep and exacerbating fatigue. Increased emotional demands likely activate the sympathetic nervous system, triggering the release of stress hormones such as cortisol and adrenaline, which impair sleep quality and disrupt physiological homeostasis [7]. These findings clarify the interrelated relationships among key variables, including the impact of emotional demands on sleep disturbance [8, 9], the effects of sleep disturbance on fatigue [13], and the combined influence of sleep disturbances and fatigue on health [13]. However, Cronbach’s alpha coefficient for emotional demands scale was relatively low, likely due to the small number of items included in the instrument. This limited internal consistency (*α* = 0.62) may have attenuated the observed relationships, thereby underestimating the true association between emotional demands and health outcomes [37]. Future research should consider incorporating validated complementary measures to strengthen the reliability of this construct.

Night shift rotation intervals did not significantly affect sleep disturbance in this study. This finding contradicts previous research showing that longer shift rotation intervals improved sleep quality in the Korean manufacturing industry [34]. This discrepancy may be due to differences in shift patterns, as participants in the earlier study had predictable, extended rotation intervals [34], whereas the nurses in this study worked irregular schedules with shorter intervals averaging 7 days. In contrast to the previous study, which compared night‐shift rotation intervals of 6 weeks, 4 weeks, and 12 days among manufacturing shift workers [34], the present study categorized the night‐shift rotation interval using a median split of 9 days. This approach may have reduced the statistical power to detect the influence of rotation intervals on sleep disturbance. These findings underscore the need for longitudinal research employing more detailed and objective measurements of shift schedules to clarify the impact of shift‐rotation patterns on sleep and health outcomes.

Third, sleep disturbance significantly affected both fatigue and health, highlighting its central role in the well‐being of night shift nurses [13]. Notably, the direct effect of sleep disturbance on health (*β* = 0.18) was stronger than its indirect effect via fatigue (*β* = 0.09), suggesting that poor sleep quality alone can harm health, even in the absence of fatigue. This finding aligns with previous research linking sleep deprivation to digestive symptoms, mental health risks, and overall health deterioration among shift workers [4, 5, 27].

These findings have important practical implications for improving the health of night shift nurses. Intervention strategies should prioritize reducing work–life interference, managing emotional demands, and addressing sleep disturbances. Cognitive‐behavioral programs that enhance coping skills for emotional demands at work, along with regular rest breaks and recovery opportunities, can help reduce work stress and improve sleep quality [38, 39]. Hospital administrators should consider implementing flexible shift scheduling that support work–life balance, such as allowing nurses to select shift types and working hours that accommodate their personal and family needs [40]. Additionally, promoting exercise programs tailored to shift nurses may help alleviate fatigue and improve overall health outcomes.

## 5. Limitations

This study has several limitations. First, the participants were predominantly young and female nurses from metropolitan areas in South Korea, which limits the generalizability of the findings to other populations, such as older nurses, male nurses, or healthcare systems in western countries. Cultural norms regarding work hours and social support in Korean hospitals may differ from those in western contexts, further limiting cross‐cultural generalizability. Second, the analysis did not consider individual circadian rhythms and other demographic characteristics. Third, although the hypothesized mediation effects were statistically supported, these relationships are correlational rather than causal because the data were cross‐sectional. The identified indirect paths should therefore be interpreted as theoretical associations, and future longitudinal research is required to verify temporal mediation mechanisms. Finally, the short data collection period may not have fully captured the long‐term effects of shift work. Future longitudinal studies with more diverse samples and extended observation periods are needed to address these limitations.

## 6. Conclusions

Maintaining work–life balance is crucial for improving the health of night shift nurses, as it directly and indirectly mitigates the negative effects of sleep disturbance and fatigue. Emotional demands harm health indirectly through these pathways, while exercise enhances health by reducing fatigue. These findings highlight the need for tailored interventions to manage emotional demands, foster work–life balance, and encourage physical activity in night shift workers. Longitudinal research is needed to further explore the causal relationships among work and nonwork factors, sleep, fatigue, and health, as well as to develop evidence‐based policies and intervention programs.

## Disclosure

This study is based on the first author’s doctoral dissertation.

## Conflicts of Interest

The authors declare no conflicts of interest.

## Author Contributions

Bo Min Jeon: conceptualization, data collection, data analysis, and writing. Su Hyun Kim: conceptualization, data collection, writing, and editing.

## Funding

This research was supported by the Basic Science Research Program through the National Research Foundation of Korea funded by the Ministry of Science, ICT, and Future Planning (NRF‐2020R1A2C110161011).

## Supporting Information

Table S1. STROBE statement: Checklist of items that should be included in reports of cross‐sectional studies.

## Supporting information


**Supporting Information** Additional supporting information can be found online in the Supporting Information section.

## Data Availability

The data that support the findings of this study are available on request from the corresponding author. The data are not publicly available due to privacy or ethical restrictions.
